# Innate Immune Signaling in the Pathogenesis of Necrotizing Enterocolitis

**DOI:** 10.1155/2013/475415

**Published:** 2013-05-23

**Authors:** David J. Hackam, Amin Afrazi, Misty Good, Chhinder P. Sodhi

**Affiliations:** ^1^Division of Pediatric Surgery, Department of Surgery, Children's Hospital of Pittsburgh and University of Pittsburgh School of Medicine, One Children's Hospital Drive, 4401 Penn Avenue, Pittsburgh, PA 15224, USA; ^2^Division of Newborn Medicine, Department of Pediatrics, Children's Hospital of Pittsburgh and University of Pittsburgh School of Medicine, One Children's Hospital Drive, 4401 Penn Avenue, Pittsburgh, PA 15224, USA

## Abstract

Necrotizing enterocolitis (NEC) is a challenging disease to treat, and caring for patients afflicted by it remains both frustrating and difficult. While NEC may develop quickly and without warning, it may also develop slowly, insidiously, and appear to take the caregiver by surprise. In seeking to understand the molecular and cellular processes that lead to NEC development, we have identified a critical role for the receptor for bacterial lipopolysaccharide (LPS) toll like receptor 4 (TLR4) in the pathogenesis of NEC, as its activation within the intestinal epithelium of the premature infant leads to mucosal injury and reduced epithelial repair. The expression and function of TLR4 were found to be particularly elevated within the intestinal mucosa of the premature as compared with the full-term infant, predisposing to NEC development. Importantly, factors within both the enterocyte itself, such as heat shock protein 70 (Hsp70), and in the extracellular environment, such as amniotic fluid, can curtail the extent of TLR4 signaling and reduce the propensity for NEC development. This review will highlight the critical TLR4-mediated steps that lead to NEC development, with a focus on the proinflammatory responses of TLR4 signaling that have such devastating consequences in the premature host.

## 1. Introduction

Necrotizing enterocolitis (NEC) is a challenging disease to treat. While NEC may develop quickly and without warning, it may also develop slowly, insidiously, and to take the caregiver by surprise. In its most severe and extreme form, NEC is not particularly difficult to recognize; the premature infant with a grossly distended abdomen, bilious nasogastric aspirates, and maroon-colored stools can be readily considered to have NEC as a unifying explanation for his/her constellation of symptoms. In earlier stages of the disease, however, where the only symptoms may be feeding intolerance and mild hemodynamic instability, the diagnosis of NEC often cannot be made with high confidence or reliability, reflective perhaps of the fact that a variety of septic states can share features that overlap with NEC. Yet despite the inability to reliably diagnose NEC in these early stages, it is precisely at the earliest stages of the disease where an accurate diagnosis is most critical, as it is here that the ability to intervene may be expected to have the greatest potential for benefit before irreversible intestinal necrosis and overwhelming systemic sepsis occur. Given the relative imprecise nature of the diagnostic approaches for early stage NEC, scientists who investigate the molecular underpinnings of this disease and clinicians who take care of patients who suffer from it have focused their attention on gaining a greater understanding of the events that lead to its early development. In this regard, we and others have identified a necessary role for the innate immune lipopolysaccharide receptor toll like receptor 4 (TLR4) in the pathogenesis of NEC. In this review, we will investigate the evidence that points to a role for TLR4 signaling in the pathogenesis of NEC, through its ability to promote intestinal injury and through its deleterious effects on intestinal healing in the premature host that together lead to the development of NEC.

## 2. Injury and Repair in the Intestinal Tract of the Premature Infant

The intestinal mucosa of the premature infant exists in a state of constant injury and repair, which must be perfectly balanced in order to maintain homeostasis. Injury to the intestinal mucosa occurs during a variety of settings that may be present within the setting of prematurity, including hypoxia [1, 2], remote infection [3], and the administration of nonbreast milk infant formula [4]. We [5] and others [6] have shown that the initial injury to the small intestine primarily involves the loss of epithelial villi through apoptosis, which subsequently leads to the development of necrosis, a process that is consistent yet only incompletely explained. Loss of the epithelial barrier through apoptosis permits the translocation of bacteria and other antigens that are present within the lumen of the intestine, and which must normally be appropriately shielded from the immune system of the premature host in order to prevent the exaggerated inflammatory response, that is, typical of intestinal inflammatory conditions such as NEC [7, 8]. In response to the loss of epithelial continuity (which may be reflective of primary apoptosis or the early events that migh culminate in apoptosis), a multipronged healing program is initiated. Healing of the intestinal mucosa occurs initially through the process of enterocyte migration, which involves the movement of healthy enterocytes into the wounded area in order to provide a rapid seal, which limits the degree of bacterial translocation that can occur [9]. While enterocyte migration can facilitate the early steps that lead to intestinal restitution, these events are short lived and unlikely to have a long-lasting effect without the generation of new enterocytes, a process that occurs within the Crypts of Luberkuhn [10, 11]. The steps that regulate the proliferation of enterocytes from existing precursors have been well characterized in a series of thorough recent reviews [12, 13]. We have described that under conditions of prematurity, NEC is associated with a marked inhibition in both enterocyte migration and proliferation, which renders the host uniquely susceptible to further injury through the combined loss of both of the important reparative processes that are normally present within the intestine [5]. In the subsequent sections, the mechanisms by which enterocyte migration and proliferation are each impaired in the pathogenesis of NEC will be reviewed in further detail.

## 3. Activation of the Innate Immune Receptor Toll Like Receptor 4 (TLR4) Inhibits Enterocyte Migration

As described above, the earliest reparative event that occurs in response to epithelial injury involves the migration of healthy enterocytes from uninjured areas to sites of epithelial disruption. We [5, 14, 15] and others [16] have shown that necrotizing enterocolitis is characterized by a marked impairment in enterocyte migration, and, as a result, severe mucosal defects are present. In seeking to identify the mechanisms by which the impairment in enterocyte migration occurs, we have shown that TLR4 is expressed within the intestinal epithelium, and that activation of TLR4 leads to an abrupt inhibition in enterocyte migration both in vivo and in vitro [14, 15]. We have further demonstrated that the reduction in enterocyte migration in response to TLR4 activation occurs via an increase in actin-mediated stress fibers, leading to an increase in the degree of adhesiveness with which the enterocyte is anchored to the underlying basement membrane [17]. The increase in stress fibers occurs due to a TLR4-dependent increase in the activity of the small GTP-ase RhoA, which catalyzes the formation of stress fibers within the cytoplasm, and through the activation of focal adhesion kinase (FAK), which further strengthens the degree of anchoring of the enterocyte to the underlying membrane [5, 15]. TLR4 signaling on enterocytes also leads to an efflux of beta-1 integrins from the cytoplasm towards the cell membrane, resulting in enhanced cell-matrix contacts that together serve to limit the degree of cell movement that can occur [18]. In support of the physiological relevance of these findings, the treatment of cells with either antibodies to beta integrins or with reagents that inhibit FAK activation can reverse the deleterious effects of TLR4 activation on enterocyte migration and promote cell movement, even in the face of tonic TLR4 signaling [5, 15, 18]. In further studies, we have shown that intercellular communication via gap junctions is required for effective enterocyte migration to occur, and that NEC is characterized by a marked reduction in the expression of gap junctions on the surface of enterocytes that is mediated through the release of the proinflammatory cytokine interferon gamma [19]. In additional studies, we showed that interferon gamma inhibited enterocyte migration through the displacement of Connexin43 from lipid rafts, which are discrete and highly organized areas of the plasma membrane that represents important sites of cellular signaling [20]. In addition, the proinflammatory signaling molecule nitric oxide was shown to play an important role in the regulation of enterocyte migration in NEC as exposure of enterocytes to nitric oxide was shown to impair cell movement both in vitro and in vivo, in part through the activation of RhoA and enhancement of cell-matrix adhesiveness [14]. Consistent with these studies, Besner and colleagues have examined the role of E-cadherin and integrins in NEC, and have determined that the growth factor heparin bound epidermal growth factor (HB-EGF) can promote intestinal restitution in NEC through effects on integrin-extracellular matrix interactions and intercellular adhesions [16]. These studies are particularly relevant, as they support an earlier observation in human patients, in which a decrease in trefoil peptides that are known to play a key role in intestinal regeneration was found to be reduced in NEC [21]. Taken together, these findings illustrate important role of impaired enterocyte migration in the pathogenesis of NEC and identify important mechanistic events in mediating the impaired migration mediating these events.

## 4. TLR4 Activation on the Intestinal Epithelium Leads to Enterocyte Apoptosis

One of the earliest changes that are observed within the intestinal mucosa in the setting of experimental NEC is a marked increase in the loss of enterocytes, which die through the process of exaggerated apoptosis [7, 21–26], leading to the transluminal passage of indigenous microbes and an unbridled activation of the host immune system. We have recently identified that the activation of toll like receptor 4 (TLR4) within the intestinal epithelium plays a critical role in the early initiation of the steps that lead to enterocyte loss [5, 27–29], a finding that is supportive of studies by Jilling and colleagues showing that mice deficient in TLR4 were protected from NEC development [30]. TLR4 signaling in enterocytes both in vitro and in vivo leads to enterocyte apoptosis, while the inhibition of TLR4 signaling in the newborn intestinal epithelium prevents NEC development and attenuates the degree of enterocyte apoptosis [5, 27, 28]. While these studies have placed the spotlight on the role of TLR4 in the pathogenesis of NEC, the observation that most premature infants do not develop NEC despite the seemingly tonic activation of TLR4 within the intestinal epithelium and elsewhere raises the important possibility that TLR4 signaling must somehow be curtailed within the newborn intestinal epithelium, in order to limit the propensity for spontaneous NEC development. This concept will be explored in detail below.

## 5. Cellular Strategies That Limit the Extent of TLR4 Signaling in the Intestinal Epithelium

In seeking to define whether negative regulatory strategies for TLR4 within the newborn intestinal epithelium could participate in the pathogenesis of NEC, we focused on the intracellular chaperone heat shock protein 70 (Hsp70), to determine whether perhaps Hsp70 could negatively regulate TLR4 signaling within enterocytes and by extension, whether a loss of Hsp70 could lead to NEC development through uninhibited TLR4 activation. The heat shock proteins—of which Hsp70 is a predominant member—represent a family of intracellular proteins that are activated by a variety of stressors and that can assist in the delivery of target proteins to the ubiquitin-proteosome system for degradation through cochaperone molecules such as CHIP, which stands for C-terminus of Hsp70 interacting protein [31]. In support of a possibility for Hsp70 in the regulation of enterocyte apoptosis, we note that TLR4 has previously been shown to play an important role in the modulation of apoptosis after various forms of stress [32–35], and Hsp70 has been shown to serve a protective role in the intestine as demonstrated by Tao and colleagues [36, 37]. Through its combined roles of both clearing proteins and modulating cell death, the net effect of Hsp70 induction within cells is to restore the host to a nonstressed environment [38–40]. We have recently determined that the expression of TLR4 is significantly reduced in mice and humans with NEC [22], suggesting but not proving that Hsp70 may negatively regulate TLR4 signaling and that a reduction in Hsp70 may result in the progression to NEC. In support of this possibility, using enterocytes that either lack or are induced to express Hsp70 as well as by examining mice that either lack Hsp70 or that overexpress Hsp70 within the intestinal epithelium, we have determined that intracellular Hsp70 limits TLR4 signaling in enterocytes and, moreover, that Hsp70 plays a central role in the pathogenesis of NEC [22]. The mechanism by which Hsp70 limits TLR4 signaling in the gut involves an increase in CHIP-mediated ubiquitination and degradation of TLR4 via the ubiquitin-proteosomal pathway [22]. Importantly, pharmacologic upregulation of Hsp70 within the intestinal mucosa led to a reduction in TLR4 signaling and a decrease in enterocyte apoptosis, leading to an attenuation in NEC severity [22]. Taken together, these findings illustrate a novel pathway linking the regulation of Hsp70 with the negative control of TLR4 signaling within the gut and provide evidence that the development of NEC results in part from exaggerated TLR4-induced enterocyte apoptosis due in part to reduced Hsp70 activity [22]. Moreover, these results suggest that pharmacologic upregulation of Hsp70 could provide a novel approach to the prevention and/or treatment of NEC through the inhibition of TLR4 signaling in the newborn intestine [22, 41]. 

It is important to note that the findings in which cytoplasmic Hsp70 serves to curtail the signaling of TLR4 within the intestinal epithelium are distinct from other studies that have focused on the extracellular role of Hsp70 and other heat shock proteins in activating the innate immune system via TLR4. For instance, Retzlaff et al. showed that the exogenous administration of Hsp70 could increase IL-1, IL-6, and TNF in cultured macrophages [42], while Wheeler et al. have shown that the extracellular exposure of Hsp70 to neutrophils from wild-type mice leads to the release of IL-8, yet this effect is not observed in neutrophils from C3H/HeJ mice that have inhibitory mutations in TLR4 [43]. Whether these results reflect the effects of heat shock proteins themselves or whether the signaling responses may be due to contaminants such as LPS which could inadvertently be present within the protein preparations as some have suggested [44–47] is beyond the scope of the current discussion although this has been carefully and extensively reviewed recently [48, 49]. In contrast to studies in the field of extracellular Hsp70 biology, the novelty and importance of the current findings lie in the newly discovered link between TLR4 and Hsp70 within the enterocyte both in vitro and in vivo, and the potential etiological relevance to the development of NEC. 

## 6. TLR4 Activation on the Intestinal Epithelium Inhibits Enterocyte Proliferation in the Pathogenesis of NEC

As described above, the early events that occur in the pathogenesis of NEC involve a loss of enterocytes through apoptosis, which leads to bacterial translocation and systemic sepsis. In response to the loss of enterocytes, the host initiates a reparative process that commences with migration of enterocytes from healthy areas towards the sites of injury and subsequently involves the proliferation of enterocytes *de novo* from precursor stem cells that are located within the intestinal crypts. We and others have examined the processes that regulate enterocyte proliferation in the newborn mucosa and in particular, have examined the pathways by which enterocyte proliferation is reduced in NEC [28, 50, 51]. Previous authors have identified a critical role for the *β*-catenin-signaling pathway in enterocyte regulation, which occurs via the upstream inhibitor GSK3*β* [52]. Importantly, we recently established a link between TLR4 and *β*-catenin, which could provide a novel explanation for the initiation and propagation of the mucosal injury seen in NEC. Specifically, we demonstrated that TLR4 activation significantly impaired enterocyte proliferation in the ileum in newborn mice as well as in cultured enterocytes [28] via the inhibition of *β*-catenin signaling. These effects were specific for newborn mice and for the ileum and were not seen in the colon or in adult mice, providing additional relevance to the pathogenesis of necrotizing enterocolitis which tends to favor the ileum. To determine the mechanisms involved, TLR4 inhibited the phosphorylation of the upstream inhibitory kinase GSK3*β*, causing *β*-catenin degradation and subsequently a decrease in proliferation and repair. Strikingly, the inhibition of enterocyte *β*-catenin signaling in NEC resulted in increased enterocyte proliferation restored and attenuated NEC severity, suggesting that strategies that can promote or enhance proliferation may have a potential therapeutic role in either the prevention or treatment of NEC [28, 53].

## 7. TLR4 Activation on Intestinal Stem Cells Leads to Their Loss through Apoptosis

As described above, the ability of the intestinal epithelium to undergo regular and rapid turnover in the face of injury is determined primarily by the activity of a discrete population of stem or progenitor cells located at the base of the intestinal crypts [54–56]. Various authors have recently identified precise and reliable markers for intestinal stem cells, which have allowed for a careful evaluation of their individual capacities to divide and differentiate, including the markers Bmi1 [57–61] and Lgr5 [62–65]. Given that the intestinal stem cells exist in close proximity to the microbial flora, it stands to reason that signaling receptors that recognize components of the flora may be present on and have effects on intestinal stem cells. Given our recent findings that TLR4 can regulate enterocyte proliferation [5], we recently explored whether TLR4 itself may be expressed on the intestinal stem cells and thus regulate their function. In support of this possibility, using flow cytometry and fluorescent in situ hybridization for the intestinal stem cell marker Lgr5, we determined that TLR4 is indeed expressed on the Lgr5-positive intestinal stem cells and that the activation of TLR4 leads to a reduced proliferation and an increase in apoptosis of the intestinal stem cells both in vivo and in vitro. This finding was not observed in mice in which we had specifically removed TLR4 from the Lgr5-positive cells, confirming the in vivo significance of this effect. To define the potential molecular mechanisms involved, TLR4 was found to inhibit intestinal stem cell proliferation and increase apoptosis via the p53-upregulated modulator of apoptosis (PUMA), as TLR4 did not affect crypt proliferation or apoptosis in intestinal stem cell cultures or in mice lacking PUMA. Furthermore, the effects of TLR4 on intestinal stem cells in vivo required TIR-domain-containing adapter-inducing interferon-*β* (TRIF) but were independent of myeloid-differentiation primary response-gene (88) (MYD88) and TNF*α*. Importantly, the inhibition of PUMA in vivo restored intestinal stem cell proliferation and reduced apoptosis in NEC, which itself was associated with reduced intestinal stem cell function [66]. The findings that NEC is associated with reduced intestinal stem cells is supportive of earlier work by Besner and colleagues, who not only showed that the intestinal stem cells and all subsequent lineages were reduced in experimental NEC, but also demonstrated that the administration of the heparin-binding epidermal growth factor (HB-EGF) could restore intestinal stem cells and therefore attenuate the severity of NEC [67]. Taken together, these findings reveal therefore that the development of NEC may reflect in part a reduction in crypt progenitor cells due to exaggerated TLR4 signaling in this compartment and raise the possibility that strategies that enhance mucosal healing through effects on the now identified TLR4-PUMA axis may be harnessed therapeutically.

## 8. TLR4 Activation in Necrotizing Enterocolitis versus Inflammatory Bowel Disease

It should be noted that the role of TLR4 in the pathogenesis of NEC in its effects on promoting injury in the small intestine may be quite distinct from the role of TLR4 in other diseases of intestinal inflammation including ulcerative colitis and Crohn's disease, in which TLR4 signaling may play a lesser or perhaps even opposite role. Various authors have carefully and convincingly demonstrated that mice lacking TLR4 have increased susceptibility to the development of colitis, suggesting that TLR4 plays a protective role in this disease [68, 69]. As we have recently discussed [8, 22], there may be several reasons to account for this apparent discrepancy. For instance, TLR4 activation leads to intestinal injury in a well-defined and physiologically relevant context, namely, the newborn small intestine. In support of this concept, we have recently demonstrated that TLR4 activation with LPS leads to increased enterocyte apoptosis in the terminal ileum of newborn mice but not adult mice, and in the small intestine but not the newborn colon [27]. Further, reports that demonstrate a protective role for TLR4 in models of colitis have typically been based upon the use of global TLR4 knockout mice, in which TLR4 signaling is disrupted in enterocytes as well as T-cells and myeloid cells. We have recently shown that TLR4 signaling within the enterocyte itself is important for the induction of intestinal injury leading to NEC, using enterally administered adenoviral constructs that bear inhibitory mutations in TLR4 whose expression is largely favored within the small bowel mucosa [28, 70]. It is therefore reasonable to conclude that the protectiveeffects attributed to TLR4 signaling in the gut by previous authors may reflect in part the mitigating effects of TLR4 signaling on other cells. In support of this possibility, we note that Fukata and colleagues have recently shown in an elegant study using chimeric mice that TLR4 signaling in colonic epithelial cells worsened intestinal inflammation [71]. These findings argue that the effects of TLR4 in the development of intestinal inflammation are strongly influenced by a variety of factors, including the effector cells involved, developmental factors, and involved region of the intestine. 

## 9. Prematurity Is Associated with Increased TLR4 Expression and Activation in the Fetal Intestine

**Figure 1:**
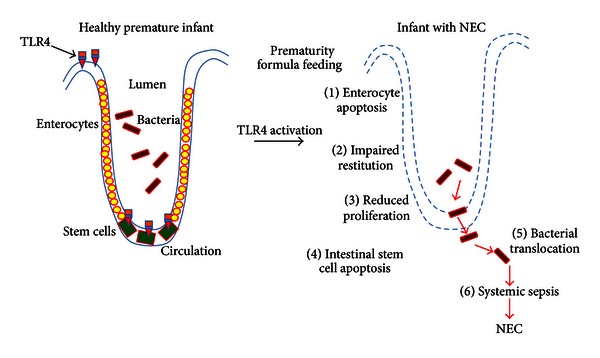
Mechanisms to explain how TLR4 activation in the premature intestinal epithelium is required in the pathogenesis of necrotizing enterocolitis. As shown, colonization of the intestine of the premature infant leads to the activation of TLR4 within the intestinal epithelium as well as on the intestinal stem cells, due in part to the elevated expression levels of TLR4 in the setting of prematurity. TLR4 activation leads to enterocyte apoptosis, impaired restitution and reduced proliferation as well as stem cell apoptosis, leading to bacterial translocation, systemic sepsis, and the development of NEC. See text for further details.

Given our findings regarding the critical importance of TLR4 signaling in enterocytes in the pathogenesis of NEC via effects on mucosal injury and repair, we next considered why the premature infant is at uniquely increased risk for the development of NEC in the first place. To do so, we considered whether the expression of TLR4 may be higher in the premature infant compared with the full-term infant and, further, whether the increased elevation of TLR4 could contribute to the development of NEC. To test this possibility directly, using quantitative RT-PCR, we determined that the expression of TLR4 is significantly greater in the premature mouse and human compared with the full-term state and moreover, the expression of TLR4 is significantly elevated under conditions that are particularly relevant to the pathogenesis of NEC, namely, the presence of hypoxia and exogenous bacteria or LPS [72, 73]. We further demonstrated that TLR4 is not only expressed at high levels within the premature intestine, but it is also functionally active [70], as the delivery of the TLR4 agonist LPS directly into the intestinal lumen of the developing mouse using ultrasound guided microinjection showed a marked increase in the induction of TLR4-dependent cytokines that matched the expression of TLR4. These findings lead us to propose that, in the setting of prematurity, TLR4 expression is very high and yet must normally decrease shortly after birth to levels that allow for the normal adaptation of the intestinal mucosa to bacteria. However, in the setting of prematurity, levels of TLR4 remain high, as we and others have shown and remain at high levels after birth in the premature state [70, 74–76]. When the premature intestine with accompanying elevated expression levels of TLR4 becomes colonized, in the setting of the correct environment that favors exaggerated TLR4 signaling, TLR4-mediated enterocyte apoptosis and delayed mucosal repair ensue, which together favor bacterial translocation and the development of systemic sepsis and NEC (see Figure 1). These findings provide additional mechanistic insights into NEC development and also provide additional insights into the increased susceptibility of the premature infant to NEC compared to full-term infants.

## 10. TLR4 Signaling in the Intestinal Epithelium Regulates the Normal Differentiation of the Intestinal Epithelium and Is Required for Goblet Cell Formation

We have recently examined the reasons for which TLR4 is significantly elevated within the intestine of the fetal mouse and human and in particular have considered the possibility that TLR4 could exert a role in the normal development of the intestine itself. To do so, we generated a novel strain of mice in which TLR4 had been selectively deleted from the intestinal epithelium and examined the differentiation of the small intestine in these mice [29]. To our surprise, we noted that mice lacking TLR4 showed an unusual phenotype that was characterized by a marked increase in goblet cells [29]. In seeking the mechanism by which TLR4 could regulate goblet cell differentiation, we further determined that deletion of TLR4 was associated with a marked decrease in signaling through the Notch pathway, which is a major determinant of differentiation within the intestinal epithelium, and that this phenotype could be reversed by overexpression of TLR4 in vitro [29]. These findings are consistent with earlier studies that had shown that Notch signaling could be activated by TLR stimulation [77] in macrophages. We also determined that exposure of bile acids to TLR4-deficient IEC-6 cells as well as to crypts from TLR4-deficient mice reversed the goblet cell phenotype, a finding that is particularly relevant given that bile acids can activate Notch in the gastrointestinal epithelium [78, 79], and that increased bile acids were associated with reduced goblet cells in newborn rats and increased NEC severity [80]. These findings together indicate that TLR4 can regulate goblet cell differentiation and suggest the possibility that bile acids may serve as intermediates in the regulation of Notch. In seeking to understand the potential teleological explanation of the regulation of intestinal differentiation by an innate immune receptor, it is apparent that TLR4 activation in the regulation of intestinal differentiation is likely distinct from its signature role in host defense. It is possible that in the postnatal environment to which the premature infant is exposed, in which the expression of intestinal TLR4 remains persistently elevated—the *developmental* role for TLR4 switches to a *proinflammatory* role upon its interaction with colonizing microbes, leading to NEC development. Indeed, we have shown a separate role for TRIF and MyD88, with the former being required for goblet cell differentiation and the latter playing a key role in proinflammatory cytokine production; the future identification of molecules that can selectively activate TRIF and not MyD88 may reveal important clues into the developmental role for TLR4 within the developing gut.

## 11. Amniotic Fluid: An Elixir That Can Regulate TLR4 Signaling within the Gut of the Developing Fetus?

The developing fetus exists in a state of persistently exaggerated TLR4 expression, due in part to our recent identification of the role for TLR4 in regulating the normal differentiation of the intestinal epithelium [29]. However, the persistently elevated expression of TLR4 during development renders the fetus at risk for potentially deleterious effects; should in fact the normally sterile environment of the womb in fact be breached by microbial pathogens. Several reports have shown that the microbial DNA may be detected within the amniotic fluid itself, raising the possibility that bacterial colonization of this normally sterile environment may occur [81, 82]. It would seem therefore that in order to limit the consequencesof exaggerated TLR4 signaling in that may occur in the fetus upon exposure to microbes, there must be a counterregulatory mechanism that could limit TLR4 activation. In addressing what the potential anti-TLR4 signaling effects could be, we focused on the fact that the fetus is continuously swallowing amniotic fluid and, as a result, the fetal intestine becomes exposed to amniotic fluid as a natural consequence of the in utero environment. This raised the possibility that perhaps amniotic fluid itself could serve to limit the extent of TLR4 signaling within the intestine. In support of this possibility, we demonstrated recently that amniotic fluid reduces TLR4 signaling in the fetal intestinal mucosa, as well as in cultured enterocytes that had been exposed to bacterial products [83], resulting in a marked reduction in the degree of proinflammatory cytokine release. In seeking the molecular mechanism involved, we focused on the fact that amniotic fluid is extremely rich in epidermal growth factor (EGF), which we showed to be required for its inhibitory effects on TLR4 signaling via its ability to activate the transcription factor peroxisome proliferator-activated receptor gamma (PPAR*γ*) and, indeed, amniotic fluid did not inhibit TLR4 signaling in enterocytes that were deficient in either the EGF receptor or in PPAR*γ*, nor in mice lacking EGFR within the intestinal epithelium [83]. Further evidence that EGF in amniotic fluid was responsible for its inhibitory effects on TLR4 signaling was found in the observation that purified EGF attenuated the exaggerated intestinal mucosal TLR4 signaling, while depletion of EGF from amniotic fluid reversed the protective effects [83]. Strikingly, the development of NEC in both mice and humans was associated with reduced expression of EGFR within the intestinal epithelium that was restored upon the administration of amniotic fluid to mice and, moreover, the administration of amniotic fluid significantly attenuated NEC severity in mice. These findings may explain how premature infants may be at increased risk for NEC development due to the lack of exposure to the protective effects of amniotic fluid in the setting of elevated TLR4 expression [83]. Given that several groups have reported that the administration of EGF or its homologue HB-EGF can treat experimental NEC [50, 84], it is tempting to speculate that these prior studies may be explained through an inhibitory effect of these growth factors on TLR4 signaling, in addition to their restorative and reparative effects on the intestinal mucosa.

## 12. Other Factors That Are Important in the Pathogenesis of Necrotizing Enterocolitis

Despite the focus on TLR4, we readily acknowledge that there are other important factors that are important in NEC pathogenesis. For instance, Besner and colleagues have shown that heparin binding epidermal growth factor (HB-EGF) is important in regulating the pathogenesis of NEC, in part through determining the extent of enterocyte migration [85]. This work is in agreement with the findings of Clark and colleagues as well as Sheng and colleagues, who have demonstrated that EGF administration can attenuate NEC severity by enhancing the healing response to mucosal injury [86, 87]. Soliman and colleagues have demonstrated that platelet-activating factor (PAF) plays an important role in NEC pathogenesis and have shown that TLR4 signaling can upregulate PAF expression and therefore increase injury in experimental NEC [88, 89]. Cherrington and colleagues have shown that the accumulation of ileal bile acids causes significant injury in the small intestine in NEC pathogenesis [90], of which we showed to act in concert with TLR4-mediated impairment of enterocyte function [29]. In a parallel line of investigation, we showed that intact intestinal restitution requires intercellular connectivity that is mediated through small channels termed gap junctions that are rich in the protein connexin 43 [19, 20]. Importantly, we have shown that proinflammatory cytokines including interferon gamma cause the internalization of connexin 43, thereby impairing intercellular connectivity and reducing the extent of intestinal restitution in the pathogenesis of NEC [19, 20]. Taken together, these lines of evidence indicate that, in addition to TLR4, there are other important factors that also play significant roles in the pathogenesis of NEC, which should be taken into account when seeking a full and complete understanding of the pathogenesis of this complex and interesting disease.

## 13. Model: TLR4 Activation Plays a Critical Role in the Pathogenesis of NEC

The studies described above lead us to propose the following model to explain how NEC develops part to exaggerated TLR4 in the intestinal mucosa of the premature infant (Figure 1). As we have recently described [91], under normal conditions, the full-term infant is characterized by low levels of TLR expression, which in the setting of microbial colonization does not elicit a particular proinflammatory response and also allows for the normal adaptation of the small intestine to the colonizing microbes. By contrast, under conditions of prematurity, TLR4 expression remains high, as a consequence in part of the developmental role of TLR4 in utero, in which TLR4 is important for the normal differentiation of the intestinal epithelium. When the premature infant intestine is colonized by flora within the neonatal intensive care unit and in the absence of host-derived factors that normally serve to restrict TLR4 signaling such as Hsp70, TLR4 signaling within the gut becomes particularly exaggerated, leading to increased mucosal injury and decreased mucosal repair. The net effect of these TLR4-mediated responses leads to gut barrier failure, bacterial translocation, the development of systemic sepsis, and the clinical and pathologic features of necrotizing enterocolitis. Based upon these findings, we propose that an understanding of the early TLR4-mediated signaling events will not only allow for a greater understanding of the pathogenesis of NEC, but also offer new and innovative approaches to its treatment based upon their capacity to inhibit TLR4 signaling within the gut. Through these efforts, it is our hope and the hope of all those involved in the care of infants with NEC that these tiny infants may one day be seen as they have never been seen before—as entirely curable.
